# A RNase H2‐Linked TaqMan‐MGB Quantitative Real‐Time PCR Assay for Differential Detection of *Mycoplasma hyopneumoniae* Live‐Attenuated Vaccine Strains

**DOI:** 10.1155/tbed/5823134

**Published:** 2026-04-30

**Authors:** Kangyao Peng, Yanna Wei, Yuzi Wu, Ting Yuan, Jiahao Li, Ping Jiang, Qingyun Xie, Zhixin Feng

**Affiliations:** ^1^ School of Veterinary Medicine, Nanjing Agricultural University, Nanjing, 210095, China, njau.edu.cn; ^2^ Institute of Veterinary Medicine, Key Laboratory of Veterinary Biological Engineering and Technology, Ministry of Agriculture, Jiangsu Academy of Agricultural Sciences, Nanjing, 210014, China, jaas.ac.cn

**Keywords:** live-attenuated vaccine, *Mycoplasma hyopneumoniae*, qPCR, RNase H2

## Abstract

*Mycoplasma hyopneumoniae* (*M. hyopneumoniae*), the etiological agent of porcine enzootic pneumonia, is one of the most prevalent and economically significant respiratory pathogens in the swine industry. Vaccination constitutes a primary strategy for controlling *M. hyopneumoniae* infections. Live‐attenuated vaccines confer protection against wild‐type *M. hyopneumoniae* through competitive exclusion of early lung colonization and induce multifaceted immune responses, including humoral, mucosal, and cellular immunity, thereby exhibiting superior protective efficacy. However, the lack of methods for differentiating live‐attenuated vaccine immunization from wild‐type infection has hindered the widespread application of live‐attenuated vaccines. In this study, we developed a quantitative real‐time polymerase chain reaction (qPCR) method based on the ribonuclease H2 (RNase H2) cleavage principle, which emits specific fluorescent signals exclusively when the probe is fully complementary to the template of the live‐attenuated vaccine strains. This qPCR method exhibited high specificity and sensitivity for the *M. hyopneumoniae* live‐attenuated vaccines (168‐L and RM48), with a lower detection limit of 10 copies/µL. Using this qPCR method, the bacterial shedding dynamics of the 168‐L vaccine strain in piglets were effectively monitored, with the first detection in the nasal cavity at 3 days postvaccination (dpv), and a peak detection rate of 100% between 10 and 17 dpv. This established assay can specifically monitor the respiratory colonization and shedding of the vaccine strain after *M. hyopneumoniae* live‐attenuated vaccine immunization, thereby providing crucial technical support for the clinical evaluation of immunization efficacy.

## 1. Introduction

*Mycoplasma hyopneumoniae* (*M. hyopneumoniae*) is the causative agent of porcine enzootic pneumonia, a chronic respiratory disease characterized by growth retardation in pigs and a key agent involved in the porcine respiratory disease complex (PRDC) [1]. Since the implementation of enhanced biosecurity measures following the initial outbreak of African swine fever (ASFV) in China in 2018, the detection rate of *M. hyopneumoniae* has increased significantly. The widespread prevalence of *M. hyopneumoniae* causes substantial economic losses to the global swine industry [2]. Currently, antibiotic therapy and vaccination are the primary strategies for clinical control [3]. Most commercial *M. hyopneumoniae* vaccines are adjuvanted inactivated whole‐cell preparations [4]. Meanwhile, *M. hyopneumoniae* live‐attenuated vaccines have been licensed for use in Mexico and China. The Mexican live vaccine is an attenuated formulation based on the LKR strain, while those in China are prepared from the 168‐L and RM48 strains [5]. To maximize the preventive efficacy of these biological products, their application must be integrated with rapid clinical diagnosis, both of which are indispensable for the effective prevention and control of *M. hyopneumoniae* epidemics.

Comparative studies on the immune efficacy of *M. hyopneumoniae* vaccine have demonstrated that live‐attenuated vaccines induce fewer lung lesions and elicit multifaceted immune responses, including humoral, mucosal, and cellular immunity. Specifically, the levels of specific secretory immunoglobulin A (sIgA) and interferon‐gamma (IFN‐γ) are directly correlated with the protective efficacy against *M. hyopneumoniae* infection [6]. Owing to these advantages, *M. hyopneumoniae* live‐attenuated vaccines are widely used in China. However, the lack of reliable differential diagnostic techniques for vaccine strains has resulted in a paucity of data on the immune status and infection characteristics of *M. hyopneumoniae* live‐attenuated vaccines under clinical conditions.

Currently, the main detection methods for *M. hyopneumoniae* include isolation and culture, serological testing, and nucleic acid assays. Although bacterial isolation and culture are considered the “gold standard” for *M. hyopneumoniae* identification, the limited biosynthetic and metabolic capabilities of *M. hyopneumoniae* restrict identification efficiency [7]. Consequently, serological and molecular etiological diagnostic methods have emerged as alternatives. Ding et al. [8] developed an immunoglobulin G enzyme‐linked immunosorbent assay (IgG‐ELISA) to distinguish convalescent sera from *M. hyopneumoniae*‐infected pigs from those immunized with inactivated vaccines. Bai et al. [9] established an sIgA‐ELISA for differentiating *M. hyopneumoniae*‐infected pigs from those immunized with an inactivated vaccine. Fourour et al. [10] developed a polymerase chain reaction (qPCR) method based on the P102 gene, which exhibited excellent detection efficacy for *M. hyopneumoniae*. Li et al. [11] established a rapid detection method for *M. hyopneumoniae* using recombinase‐aided amplification (RAA) combined with the CRISPR/Cas12a system. However, no molecular differential diagnostic method currently exists to distinguish between naturally infected and live‐attenuated vaccine‐immunized animals for *M. hyopneumoniae*.

Therefore, this present study aims to establish a specific and sensitive method for the differential detection of *M. hyopneumoniae* live‐attenuated vaccine strains (168‐L and RM48). This method will serve as a molecular tool for the clinical evaluation of immunization efficacy of *M. hyopneumoniae* live‐attenuated vaccines, enabling the specific detection of vaccine strain colonization and shedding following immunization.

## 2. Materials and Methods

### 2.1. Bacterial Strains and Viruses

The live‐attenuated vaccine strains 168‐L and wild‐type strain 168 were preserved by the Institute of Veterinary Medicine, Jiangsu Academy of Agricultural Sciences. The live‐attenuated vaccine RM48 was purchased from Jilin Zhengye Biological Products Co., Ltd. The following bacterial strains and viruses were all identified and preserved by the Institute of Veterinary Medicine, Jiangsu Academy of Agricultural Sciences, including *Mycoplasma hyorhinis* (*M. hyorhinis*), *Mycoplasma hyosynoviae* (*M. hyosynoviae*), *Mycoplasma flocculare* (*M. flocculare*), *Mycoplasma bovis* (*M. bovis*), *Mycoplasma canis* (*M. canis*), *Mycoplasma gallisepticum* (*M. gallisepticum*), *Mycoplasma synoviae* (*M. synoviae*), *Mycoplasma pneumoniae* (*M. pneumoniae*), *Mycoplasma genitalium* (*M. genitalium*), *Actinobacillus pleuropneumoniae* (*A. pleuropneumoniae*), *Haemophilus parasuis* (*H. parasuis*), *Escherichia coli* (*E. coli*), *porcine circovirus* (PCV), and *porcine reproductive and respiratory syndrome virus* (PRRSV). The *M. hyopneumoniae* clinical isolates (*n* = 38) were obtained from lung tissue samples of diseased pigs showing respiratory symptoms in Guangxi, Guangdong, Anhui, and Jiangsu provinces from 2019 to 2023, were identified by universal qPCR (Supporting Information Table S1 and Figure S1), and preserved by the Institute of Veterinary Medicine, Jiangsu Academy of Agricultural Sciences.

### 2.2. Bacterial Culture and Genomic DNA Extraction

All *M. hyopneumoniae* strains were inoculated into KM2 medium at a ratio of 1:10 and cultured at 37°C for 48 h. All genomic DNA was extracted using the E.Z.N.A DNA Kit (Omega Co., Ltd.) in accordance with the instructions.

### 2.3. Genomic Comparison

Live vaccine strain 168‐L was an attenuated strain obtained by culturing wild‐type strain 168 in KM2 medium for more than 300 passages [12]. Through the whole‐genome comparison of the wild‐type parent strain and the live‐attenuated vaccine strain, differential genes were screened as candidate targets for the differential detection of the live‐attenuated vaccine strain (Supporting Information Table S2). Then, specific primers (Supporting Information Table S3) for the selected candidate targets were designed to amplify the live‐attenuated vaccine strain RM48, and the amplification products were sent to Novogene Co., Ltd. for sequencing. The homology of the candidate target genes with the RM48 strain and all 18 fully assembled *M. hyopneumoniae* genomes published on the National Center for Biotechnology Information (NCBI) was compared using TBTools‐II v2.331 and SnapGene 6.0.2. Finally, the differential sequence that specifically exists in the *M. hyopneumoniae* live‐attenuated vaccine strain was screened as the detection target.

### 2.4. Primer Design and Construction of Standard Plasmids

The probe targets a 44‐bp region corresponding to nucleotide positions 1–44 of the MHP168L_314 gene in the *M. hyopneumoniae* 168‐L genome, with primers designed in the flanking regions to amplify the surrounding fragments. The target sequence was cloned into the pUC57 plasmid as a standard plasmid and synthesized by General Biology Co., Ltd., named pUC57‐vaccine. As the target sequence in individual wild‐type strains only has a single nucleotide variation (SNV) compared to the live‐attenuated vaccine strains, RNA replacement modification was introduced at the SNV site corresponding to the fifth nucleotide of the probe‐binding region, based on the specific cleavage characteristics of ribonuclease H2 (RNase H2) on RNA/DNA. The probe was labeled with a 5′ FAM reporter dye and a 3′ nonfluorescent quencher (NFQ) coupled with a minor groove binder (MGB) moiety. All primers and probes were synthesized by General Biology Co., Ltd., and the sequence information is shown in Table 1.

**Table 1:** Sequences of primers and probe.

Primer	Sequences (5′–3′)	Amplification size (bp)
F	AGACAGTGGAATCAAAATCATA	192
R	CGAAGGTATTCGGAGTGTTTA	
P	FAM‐CCAAUTGAATTTATTGGATAAAT‐NFQ‐MGB	

### 2.5. Establishment and Optimization of the Differential qPCR Method

The basic reaction system consists of 10 µL Takara Taq HS Perfect Mix (Takara Bio Inc., Japan), 0.02 U/µL of the RNase H2 Enzyme Kit (Integrated DNA Technologies, Coralville, IA, USA), 0.9 µM of the primer (F, R), 0.4 µM of the probe P, and 2 µL of the DNA sample. Deionized water was added to bring the reaction mixture volume to 20 µL. The genomic DNA of the wild‐type strain 168, the live‐attenuated vaccine strains 168‐L and RM48 were used as reference substances. The amplification reaction was carried out on the QuantStudio 5 real‐time PCR system (ThermoFisher Co., Ltd.) with automatic baseline and threshold setting [13]. The amplification reaction was performed under the following conditions: predenaturation at 95°C for 30 s, 45 cycles of denaturation at 95°C for 10 s, annealing at 60°C for 30 s. The FAM fluorophore and NFQ‐MGB quencher were selected for signal acquisition. To determine the optimal concentration in probe P, a concentration gradient was established with five experimental groups: 0.4, 0.45, 0.5, 0.55, and 0.60 µM. To optimize the annealing temperature, an annealing temperature gradient was established with five experimental groups: 58, 59, 60, 61, and 62°C. The amplification curve was plotted using GraphPad Prism 10.

### 2.6. Specificity Test

The genomic DNA extracted from vaccine strains 168‐L and RM48, wild‐type strain 168, other pathogens (*M. hyorhinis*, *M. hyosynoviae*, *M. flocculare*, *M. bovis*, *M. canis*, *M. gallisepticum*, *M. synoviae*, *M. pneumoniae*, *M. genitalium*, *A. pleuropneumoniae*, *H. parasuis*, *E. coli*, PCV, and PRRSV), and the genomic DNA extracted from clinical isolates divided into eight groups (the isolates were randomly divided into eight groups, with five isolates per group except for group 8, which contained three isolates) were used to test the specificity of the RNase H2‐linked TaqMan‐MGB qPCR method established in this study. The standard plasmid pUC57‐vaccine was used as the positive control, and enzyme‐free sterilized water was used as the blank control. The amplification curve was plotted using GraphPad Prism 10.

Additionally, the genomic DNA extracted from clinical isolates was sent to Novogene Co., Ltd. for sequencing of the genomic framework map. After uploading the genomic framework map to the PubMLST database (PubMLST—Public databases for molecular typing and microbial genome diversity), we conducted the multilocus sequence typing (MLST) analysis on the sequencing results based on the *adk*, *ropB*, and *tpiA* genes [14].

### 2.7. Sensitivity Test

The standard plasmid pUC57‐vaccine was continuously diluted with enzyme‐free sterilized water at a ratio of 10 times from 1 × 10^8^ copies/µL down to 1 copy/µL for the analytical sensitivity test of the RNase H2‐linked TaqMan‐MGB qPCR method established in this study. After three repeated experiments, the live‐attenuated vaccine strains 168‐L and RM48 of 10^9.5^ color changing unit 50%/mL (CCU_50_/mL) were continuously diluted with KM2 medium at a ratio of 10 times [15]. The genomic DNA extracted from 100 µL of each diluted sample was used to evaluate the detection sensitivity of this qPCR method. After three repeated experiments, linear regression analysis and the coefficient of determination (*R* ^2^) were performed using GraphPad Prism 10.

### 2.8. Reproducibility Test

The genomic DNA of live‐attenuated vaccine strains 168‐L and RM48, and the standard plasmid pUC57‐vaccine were used as reaction templates. The repeatability of the RNase H2‐linked TaqMan‐MGB qPCR method was tested by 10 replicate tests. The coefficient of variation (CV) was calculated by $\text{CV}\left(\%\right)=\frac{\text{SD}}{\overset{―}{x}}\times 100\%$ (SD: standard deviation, $\overset{―}{x}$: mean cycle threshold [Ct] value) and was plotted using GraphPad Prism 10.

### 2.9. Application of the Differential qPCR Method

By detecting the nucleic acid of nasal swab samples from pigs immunized with the *M. hyopneumoniae* live‐attenuated vaccine, the clinical performance of the established qPCR method was evaluated. A total of 20 piglets (7 days old) born from sows presenting clinical symptoms of *M. hyopneumoniae* infection and testing positive for *M. hyopneumoniae* antibodies were selected. They were randomly divided into two groups for an immunization trial conducted at a *M. hyopneumoniae*‐positive farm. A total of 10 piglets were housed in one pen as the vaccination group, while the other 10 piglets were housed in a separate pen as the nonvaccination control group. For the vaccination group, the piglets were vaccinated via intrapulmonary injection according to the instruction: briefly, a live‐attenuated vaccine strain 168‐L (2 × 10^9^ CCU_50_/vial) was reconstituted by diluting one vial of lyophilized vaccine in 20 mL of sterile phosphate‐buffered saline (PBS) to achieve a final concentration of 1 × 10^8^ CCU_50_/dose. Each piglet in the vaccination group received a 1 mL dose via intrapulmonary injection.

To evaluate the immunization efficacy of *M. hyopneumoniae* live‐attenuated vaccines, nasal swabs were collected longitudinally from both groups at 0, 3, 7, 10, 14, 17, and 21 days postvaccination (dpv). Total genomic DNA extracted from the nasal swab samples was used as templates for the RNase H2‐linked qPCR assay.

## 3. Result

### 3.1. Screening of Specific Molecular Target for *M. hyopneumoniae* Live‐Attenuated Vaccines

**Figure 1:**
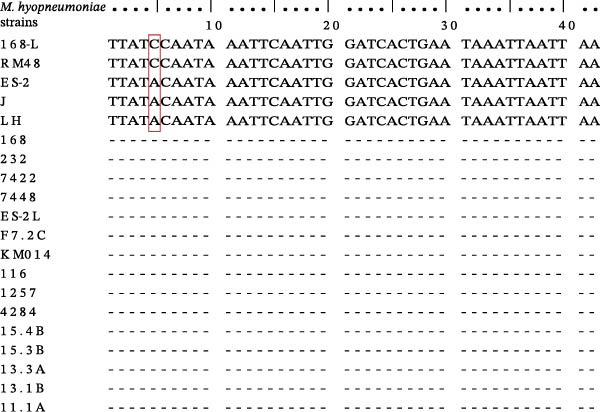
Multiple sequence alignment analysis of the target region in the MHP168L_314 gene of *M. hyopneumoniae* live‐attenuated vaccine strains and wild‐type strains.

Comparative genomics studies of *M. hyopneumoniae* wild‐type strain 168 and its live‐attenuated vaccine derivative strain 168‐L revealed that the genome of strain 168‐L is 4483 bp smaller than that of its parental strain 168, with genetic variations including 60 insertions, 43 deletions (indels), and 277 SNVs [16]. Among the 60 insertions, continuous base repeat sequences and misaligned insertion fragments were excluded, and 7 genes with longer differential sequences were ultimately selected as candidate targets (Supporting Information Table S2). Homology comparisons of these seven candidate target genes across all 18 fully assembled *M. hyopneumoniae* genomes available in the NCBI database demonstrated that the MHP168L_314 gene of the live‐attenuated vaccine strain 168‐L does not share 100% identity with corresponding genes in other strains. Except for *M. hyopneumoniae* strains J, ES‐2, and LH, which exhibited 97.73% sequence identity to the MHP168L_314 gene, no homologous sequences of this gene were detected in the genomes of other wild‐type strains (Supporting Information Table S4). Multiple sequence alignment analysis of the target region in the MHP168L_314 gene indicated that the 97.73% identity resulted from a single‐base substitution (“C” to “A”) at 5th nucleotide position (Figure 1).

### 3.2. Development and Optimization of the RNase H2‐Linked qPCR Assay

**Figure 2:**
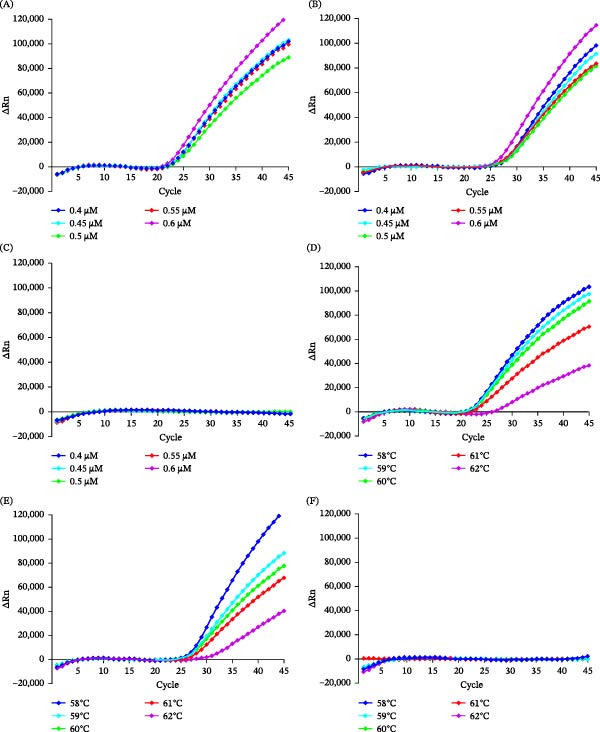
The optimization results of the RNase H2‐linked qPCR assay. (A) Probe concentration gradient (0.4/0.45/0.5/0.55/0.6 µM) for the 168‐L strain. (B) Probe concentration gradient (0.4/0.45/0.5/0.55/0.6 µM) for the RM48 strain. (C) Evaluation of nonspecific amplification of the wild‐type strain 168 under different probe concentrations (0.4/0.45/0.5/0.55/0.6 µM). (D) Annealing temperature gradient (58/59/60/61/63°C) for the 168‐L strain. (E) Annealing temperature gradient (58/59/60/61/63°C) for the RM48 strain. (F) Evaluation of nonspecific amplification of the wild‐type strain 168 under different annealing temperatures (58/59/60/61/63°C).

Optimization of probe P concentration identified 0.60 µM as the optimal working concentration. At this concentration, specific amplification was observed for the live‐attenuated vaccine strains 168‐L and RM48, with the highest fluorescence intensities detected (Figure 2A,B).

The optimal annealing temperature was determined to be 58°C, as this temperature enabled specific amplification of the 168‐L and RM48 vaccine strains with maximal fluorescence output (Figure 2D,E).

For optimization of the qPCR assay, the wild‐type strain 168 was tested under different probe concentrations (0.4, 0.45, 0.5, 0.55, and 0.6 µM) and annealing temperatures (58, 59, 60, 61, and 62°C) to ensure that the established assay would not produce nonspecific amplification signals for the wild‐type strain. No amplification signal was observed for strain 168 across the tested probe concentrations (Figure 2C) and annealing temperatures (Figure 2F).

### 3.3. Specificity Test of the RNase H2‐Linked qPCR Assay

**Figure 3:**
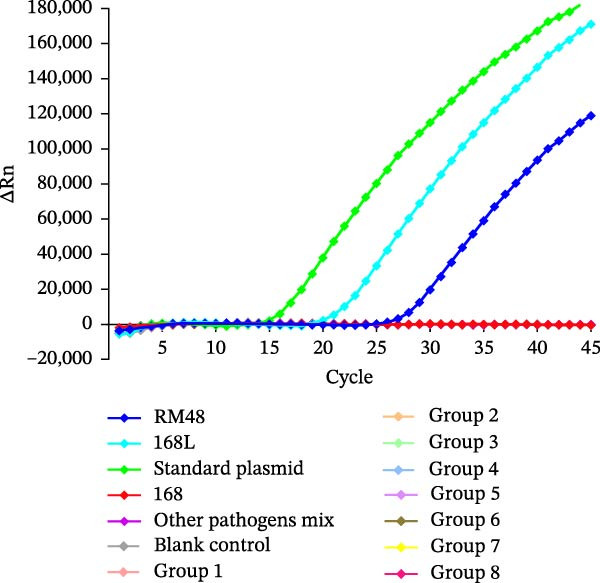
Specificity validation of the RNase H2‐linked qPCR assay using live‐attenuated vaccine strains, the wild‐type strain, other pathogens, and *M. hyopneumoniae* clinical isolates. The “other pathogens mix” includes 14 swine pathogens (*M. hyorhinis*, *M. hyosynoviae*, *M. flocculare*, *M. bovis*, *M. canis*, *M. gallisepticum*, *M. synoviae*, *M. pneumoniae*, *M. genitalium*, *A. pleuropneumoniae*, *H. parasuis*, *E. coli*, PCV, and PRRSV). Groups 1–8 represent the 38 *M. hyopneumoniae* clinical isolates, which were randomly divided into eight groups (five isolates per group, except Group 8 containing three isolates).

To validate the specificity of the RNase H2‐linked qPCR assay, genomic DNA extracted from live‐attenuated vaccine strains (168‐L, RM48), the wild‐type strain 168, other pathogenic strains (*n* = 14; including *M. hyorhinis*, *M. hyosynoviae*, *M. flocculare*, *M. bovis*, *M. canis*, *M. gallisepticum*, *M. synoviae*, *M. pneumoniae*, *M. genitalium*, *A. pleuropneumoniae*, *H. parasuis*, *E. coli*, PCV, and PRRSV), and *M. hyopneumoniae* clinical isolates (*n* = 38) randomly divided into eight groups (Supporting Information Table S5) were used as templates. The results demonstrated that specific FAM fluorescence signal amplification was exclusively observed for the live‐attenuated vaccine strains 168‐L and RM48, with no cross‐reactivity detected against the wild‐type strain 168, other pathogenic strains, or the clinical isolates (Figure 3). These findings confirm the high specificity of the established RNase H2‐linked qPCR assay.

Notably, although the RNase H2‐linked qPCR assay yielded negative results for all the aforementioned *M. hyopneumoniae* clinical isolates, MLST results indicated that isolates AH‐F21 and WX‐F14 (Group 1) shared an identical allele profile to that of the live‐attenuated vaccine reference strain 168‐L, specifically *adk* (16), *rpoB* (15), and *tpiA* (34) (Supporting Information Table S5). These inconsistent results indicate that RNase H2‐linked qPCR exhibits superior discriminatory capability compared to MLST for the specific detection of *M. hyopneumoniae* live vaccine strains.

### 3.4. Sensitivity Test of the RNase H2‐Linked qPCR Assay

**Figure 4:**
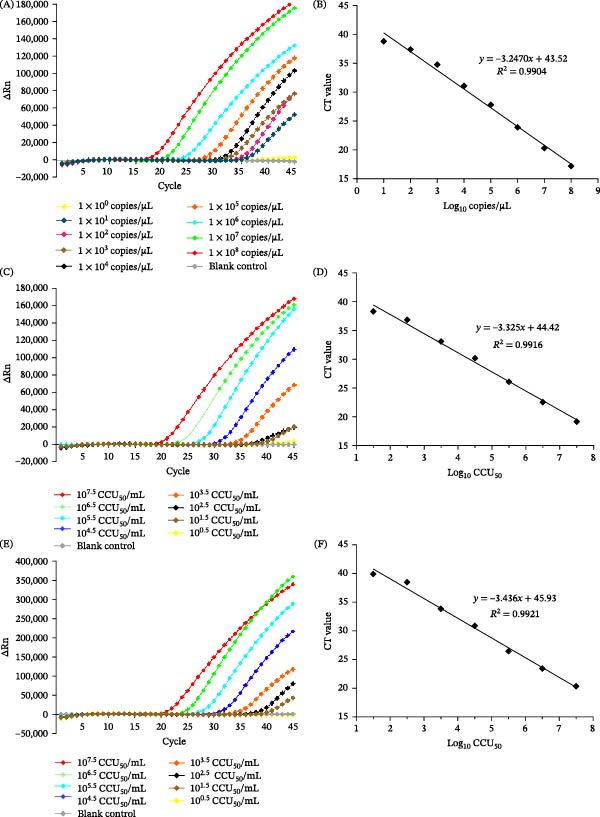
Sensitivity validation of the RNase H2‐linked differential qPCR assay. (A) Amplification curves of the standard plasmid pUC57‐vaccine at serial concentrations: 1 × 10^8^ copies/µL, 1 × 10^7^ copies/µL, 1 × 10^6^ copies/µL, 1 × 10^5^ copies/µL, 1 × 10^4^ copies/µL, 1 × 10^3^ copies/µL, 1 × 10^2^ copies/µL, 10 copies/µL and 1 copy/µL. (B) Standard curve of the pUC57‐vaccine plasmid, plotted as log_10_ copies/µL versus cycle threshold (Ct) value. (C) Amplification curves of the live‐attenuated vaccine strain 168‐L genomic DNA at serial dilutions: 10^0.5^ CCU_50_/mL, 10^1.5^ CCU_50_/mL, 10^2.5^ CCU_50_/mL, 10^3.5^ CCU_50_/mL, 10^4.5^ CCU_50_/mL, 10^5.5^ CCU_50_/mL, 10^6.5^ CCU_50_/mL, and 10^7.5^ CCU_50_/mL. (D) Standard curve of strain 168‐L, plotted as log_10_CCU_50_ versus Ct value. (E) Amplification curves of the live‐attenuated vaccine strain RM48 genomic DNA at serial dilutions: 10^0.5^ CCU_50_/mL, 10^1.5^ CCU_50_/mL, 10^2.5^ CCU_50_/mL, 10^3.5^ CCU_50_/mL, 10^4.5^ CCU_50_/mL, 10^5.5^ CCU_50_/mL, 10^6.5^ CCU_50_/mL, and 10^7.5^ CCU_50_/mL. (F) Standard curve of strain RM48, plotted as log_10_CCU_50_ versus Ct value.

To evaluate the analytical sensitivity of the RNase H2‐linked qPCR assay, 10‐fold serial dilutions of the standard plasmid pUC57‐vaccine (ranging from 1 × 10^8^ copies/µL to 1 copy/µL) were tested. As shown in Figure 4A, specific amplification was detected across all tested concentrations, with a lower limit of detection determined to be 10 copies/µL. No amplification signal was observed at 1 copy/µL. The standard curve generated from log_10_‐transformed plasmid copy numbers and corresponding Ct values exhibited a linear correlation (*y* = −3.2470*x* + 43.52, *R* ^2^ = 0.9904; Figure 4B), confirming the assay’s reliability for quantitative detection.

For the diagnostic sensitivity assessment using live‐attenuated vaccine strains, 10‐fold serial dilutions of genomic DNA from strains 168‐L and RM48 (original stock: 10^9.5^ CCU_50_/mL) were analyzed. Amplification curves for strains 168‐L and RM48 were observed for all dilutions down to 10^1.5^ CCU_50_/mL (Figure 4C,E), indicating a lower limit of detection of 10^1.5^ CCU_50_/mL for both vaccine strains. No amplification signal was observed at 10^0.5^ CCU_50_/mL. The standard curves for strains 168‐L (*y* = −3.325*x* + 44.42, *R* ^2^ = 0.9916; Figure 4D) and RM48 (*y* = −3.436*x* + 45.93, *R* ^2^ = 0.9921; Figure 4F) also showed excellent linearity, validating the assay’s ability to quantify live vaccine strain loads accurately.

### 3.5. Repeatability Test of the RNase H2‐Linked qPCR Assay

**Figure 5:**
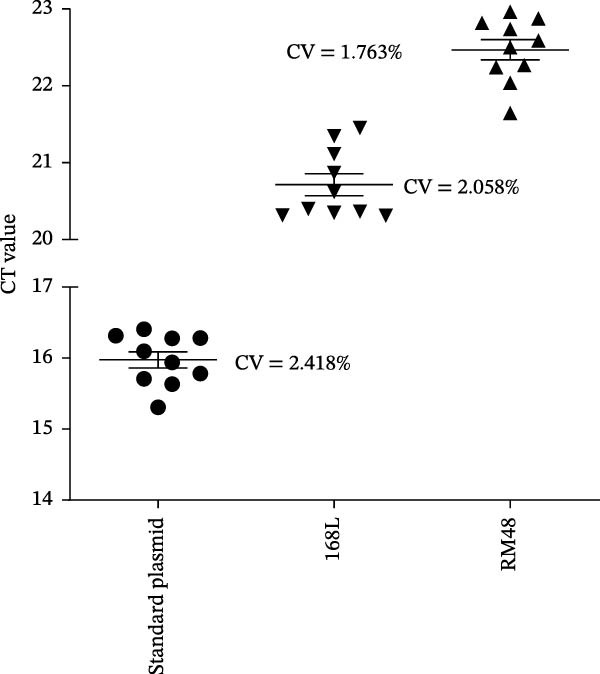
Repeatability validation of the RNase H2‐linked qPCR assay: Ct value distribution and coefficient of variation (CV) for the standard plasmid pUC57‐vaccine, 168‐L, and RM48 (10 technical replicates per template).

The repeatability of the RNase H2‐linked qPCR assay was evaluated using the standard plasmid pUC57‐vaccine and the genomic DNA from live‐attenuated vaccine strains 168‐L and RM48. For each template, the qPCR reaction was performed in 10 technical replicates under the optimized conditions. The CV values for the Ct values were calculated to assess repeatability. As shown in Figure 5, the CV values were determined to be 2.418% for the standard plasmid pUC57‐vaccine, 2.058% for strain 168‐L, and 1.763% for strain RM48. All CV values were well below the widely accepted threshold of 5% for qPCR assays, confirming that the established RNase H2‐linked qPCR method exhibits excellent stability and reproducibility for the specific detection of *M. hyopneumoniae* live‐attenuated vaccine strains.

### 3.6. Application of the RNase H2‐Linked qPCR Method

**Figure 6:**
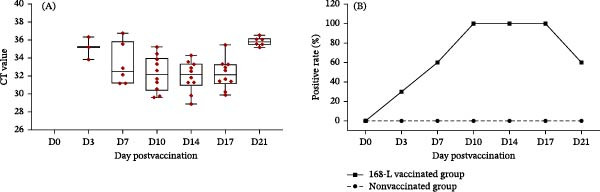
Specific detection of shedding dynamics of *M. hyopneumoniae* live‐attenuated vaccine strains 168‐L postvaccination. (A) Ct value distribution of the 168‐L vaccine strain in nasal swabs of immunized piglets at each dpv. (B) Positive rates of the 168‐L vaccinated group compared to the nonvaccinated group at each dpv.

The shedding dynamics of the live‐attenuated vaccine strains 168‐L were monitored using the RNase H2‐linked qPCR assay with nasal swabs collected longitudinally from 168‐L vaccine‐immunized and nonimmunized pigs over a 21‐day period. From 3 to 21 dpv, the detection rates were as follows: 3/10 at 3 dpv, 6/10 at 7 dpv, 10/10 at 10 dpv, 10/10 at 14 dpv, 10/10 at 17 dpv, and 6/10 at 21 dpv (Figure 6A). In the immunized group, the bacterial shedding dynamics of the 168‐L vaccine strain in piglets were effectively monitored, with the first detection in the nasal cavity at 3 dpv and a peak detection rate of 100% between 10 and 17 dpv (Figure 6B). In contrast, no positive signals were detected in the nonimmunized group throughout the entire experimental period (Figure 6B).

## 4. Discussion

*M. hyopneumoniae* is one of the most prevalent pathogens contributing to porcine respiratory diseases. It damages the mucosal epithelial system of the respiratory tract, making the infected pigs more susceptible to other respiratory pathogens such as PCV and PRRSV [17]. Vaccination remains the primary strategy for preventing and controlling *M. hyopneumoniae* infections, with inactivated vaccines being the most widely used formulation [17]. While inactivated vaccines can improve daily weight gain and reduce lung lesions in vaccinated herds, they fail to prevent *M. hyopneumoniae* colonization. In contrast, live‐attenuated vaccines confer protection by competitively occupying target organs early in infection and inducing multifaceted immune responses, including humoral, mucosal, and cellular immunity [18]. Despite these superior protective advantages, the lack of specific differential diagnostic tools has restricted postimmunization efficacy evaluation of live‐attenuated vaccines based on pathogen load quantification. Existing universal *M. hyopneumoniae* qPCR methods often misclassify piglets vaccinated with live‐attenuated vaccines as naturally infected, while published serological methods can only differentiate *M. hyopneumoniae*‐infected from inactivated vaccine‐immunized pigs [8]. Thus, the specific differential detection assay for *M. hyopneumoniae* live‐attenuated vaccines established in this study holds significant implications for the prevention and purification of porcine enzootic pneumonia.

Based on comparative genomics analysis, the MHP168L_314 gene of *M. hyopneumoniae* strain 168‐L was screened as the specific target for qPCR development. Since the gene does not share 100% sequence homology with other *M. hyopneumoniae* strains, it inherently ensures the specificity of the established method. However, wild‐type strains J, ES‐2, and LH have only a SNV site in the target sequence compared to the live‐attenuated vaccine strain (Supporting Information Table S4 and Figure S1), which could substantially reduce probe recognition specificity and lead to false‐positive results (i.e., misclassifying these wild‐type strains as vaccine strains). For SNV‐specific amplification reaction, the position of probe‐template mismatches is a critical determinant of amplification efficiency: mismatches at the 3′‐terminal significantly impair efficiency, while those at more distal positions are better tolerated [19]. The TaqMan‐MGB probe strategy is a widely used method for SNV genotyping; however, it fails to effectively eliminate nonspecific amplification of high‐concentration wild‐type templates containing SNVs (data not shown). To enhance the probe’s SNV discrimination capability, we further modified the SNV site with ribonucleotide substitution based on the TaqMan‐MGB probe design principle (Table 1). When the probe perfectly complements the target DNA sequence (including the SNV site), a DNA/RNA heteroduplex is formed. RNase H2 then specifically recognizes and hydrolyzes the ribonucleotide bond within this hybrid, resulting in probe fragmentation and the generation of a fluorescent signal detectable by real‐time PCR system. Importantly, the Taq polymerase used in the reaction system lacks 5′–3′ exonuclease activity to avoid false positive interference [20]. The RNase H2‐based molecular diagnostic strategies have been successfully applied to SNP (single nucleotide polymorphism) discrimination: Meng et al. [21] combined RNase H2‐mediated SNP recognition with loop‐mediated isothermal amplification (LAMP) to distinguish different serotypes of *Streptococcus suis* based on single nucleotide differences, demonstrating improved specificity compared to traditional LAMP assays; Tso et al. [22] combined RNase H2‐dependent PCR (rhPCR) with SNP‐based detection to differentiate pathotypes of the plant pathogen *Plasmodiophora brassicae*, demonstrating the effectiveness of RNase H2‐mediated SNP recognition for pathogen variant identification. Thus, RNase H2 serves as the key enzyme for signal activation in RNase H2‐linked qPCR, ensuring reaction specificity through strict reliance on perfect probe‐target complementarity and enabling highly specific amplification of live‐attenuated vaccine strains.

Specificity tests confirmed that the RNase H2‐linked qPCR assay exclusively amplified the *M. hyopneumoniae* live‐attenuated vaccines 168‐L and RM48, with no cross‐reactivity observed against other pathogens or *M. hyopneumoniae* clinical isolates (Figure 3). The vaccine‐associated SNV targeted by this assay is absent in clinical isolates AH‐F21 and WX‐F14 despite their MLST similarity to the vaccine strain. This may be attributed to the fact that MLST is based on housekeeping genes and is primarily used for population typing and epidemiological investigations [23], rather than for distinguishing specific mutations associated with vaccine strains. Sequence comparison of the MHP168L_314 gene from 38 clinical isolates confirmed that all clinical isolates are identical to the wild‐type strains with an adenine (A) at the SNV site, while the vaccine strains possessed cytosine (C) (Supporting Information Figure S2). In addition to strict specificity, high sensitivity is essential to avoid missed detections in practical applications. Analytical sensitivity testing revealed a lower limit of detection of 10 copies/µL for the RNase H2‐linked qPCR, which is comparable to that of a universal qPCR method targeting the conserved sequence of the P46 gene (10 copies/µL) [24]. For *M. hyopneumoniae* cultures, the limit of detection was as low as 10^1.5^ CCU_50_/mL. Repeatability testing demonstrated high stability of the method, with CV values of 2.058% and 1.763% for positive control strains 168‐L and RM48, respectively, both well within the acceptable CV range of less than 5%.

In this study, the clinical application efficacy of the established qPCR method was evaluated via a high‐dose immunization experiment using the *M. hyopneumoniae* live vaccine 168‐L. The immunization dose was set at 10‐fold the standard clinical dose to meet the safety evaluation criteria for vaccines [25]. The vaccine shedding dynamics shown in Figure 6 provide critical practical references for the prevention and control of *M. hyopneumoniae* in large‐scale swine farms. First, despite the high infection pressure in the *M. hyopneumoniae*‐positive farm, only the *M. hyopneumoniae* live‐attenuated vaccine‐immunized group tested positive for the vaccine strain, while the nonimmunized control group remained entirely negative. This result underscores the excellent specificity of the RNase H2‐linked qPCR assay for the *M. hyopneumoniae* live vaccine, which can effectively avoid interference from field strain infections and thus enable accurate analysis of the shedding dynamics of the live‐attenuated vaccine after immunization. Furthermore, the 100% detection rate of the 168‐L vaccine strain between 10 and 17 dpv not only confirms successful mucosal colonization but also establishes a key temporal reference for clinical immunization evaluation. Notably, due to the higher immunization dose, although the live vaccine strain remained detectable in pigs at 21 dpv, the corresponding Ct values increased significantly (>35). In conclusion, in practical production, veterinary technicians can conduct random nasal swab sampling and detection using the RNase H2‐linked qPCR assay approximately 2 weeks postvaccination to monitor the herd‐level immunization status and colonization intensity.

## 5. Conclusion

In this study, a TaqMan‐MGB qPCR method based on the RNase H2 enzyme cleavage principle was successfully established. A key advantage of this developed method is its ability to preclude nonspecific amplification during SNV detection, thereby enabling the specific amplification of only the live‐attenuated vaccine strains 168‐L and RM48. This method fills the gap in specific molecular detection tools for *M. hyopneumoniae* live‐attenuated vaccines and provides technical support for differentiating vaccination from wild‐type infection and evaluating the efficacy of *M. hyopneumoniae* live‐attenuated vaccines. However, the RNase H2‐linked qPCR assay demonstrated high specificity and sensitivity in this study, the limited number of clinical samples tested also presents potential limitations. Future studies incorporating more epidemiologically diverse strains of *M. hyopneumoniae* will further support the applicability of this assay in clinical diagnostics and epidemiological surveillance.

## Author Contributions

Kangyao Peng established and validated the methodology and drafted the manuscript. Yanna Wei, Yuzi Wu, and Ting Yuan were responsible for the collection, isolation, and identification of clinical strains. Jiahao Li provided funding support. Ping Jiang performed the formal analysis and provided guidance and suggestions. Qingyun Xie contributed to conceptualization, experimental guidance, and funding acquisition. Zhixin Feng contributed to conceptualization, experimental guidance, and project supervision.

## Funding

This study was supported by the National Natural Science Foundation of China (Grant 32202812) and the Jiangsu Province Association for Youth Science and Technology Talent Support Project (Grant JSTJ‐2025‐093).

## Ethics Statement

All procedures for the collection of pig samples in this study were in accordance with the protocols approved by the Ethical Committee for Animal Experiments of Jiangsu Academy of Agricultural Sciences, China (Approval ID: IACUC‐LE‐2024‐02‐001). All the pig samples were provided by the Institute of Veterinary Medicine, Jiangsu Academy of Agricultural Sciences.

## Conflicts of Interest

The authors declare no conflicts of interest.

## Supporting Information

Additional supporting information can be found online in the Supporting Information section.

## Supporting information


**Supporting Information** Figure S1: Diagnosis of clinical isolates in accordance with the Chinese Entry‐Exit Inspection and Quarantine Industry Standard (SN/T4104‐2015). Figure S2: Multiple sequence alignment of the 44 bp target region within the MHP168L_314 gene among M. hyopneumoniae live‐attenuated vaccine strains and 38 clinical isolates. Table S1: Primers and probes used in the Chinese Entry‐Exit Inspection and Quarantine Industry Standard (SN/T4104‐2015). Table S2: Seven candidate targets identified from comparative genomics analysis of M. hyopneumoniae wild‐type strain 168 and live‐attenuated vaccine strain 168‐L. Table S3: Specific primers targeting selected candidate genes for amplification of M. hyopneumoniae live‐attenuated vaccine strain RM48. Table S4: Homology comparison results of candidate genes across all 18 fully assembled M. hyopneumoniae genomes published in NCBI and RM48. Table S5: The information about the 38 clinical isolates of M. hyopneumoniae.

## Data Availability

The data that support the findings of this study are available from the corresponding author Qingyun Xie upon reasonable request.
