# Supplementary material for: A RNase H2‐Linked TaqMan‐MGB Quantitative Real‐Time PCR Assay for Differential Detection of Mycoplasma hyopneumoniae Live‐Attenuated Vaccine Strains
Source: Transbound Emerg Dis. 2026 Apr 30;2026:5823134. doi: 10.1155/tbed/5823134 (PMC13129502; doi:10.1155/tbed/5823134)
Supplement: Supplementary file 1 — Supporting Information Figure S1: Diagnosis of clinical isolates in accordance with the Chinese Entry‐Exit Inspection and Quarantine Industry Standard (SN/T4104‐2015). Figure S2: Multiple sequence alignment of the 44 bp target region within the MHP168L_314 gene among M. hyopneumoniae live‐attenuated vaccine strains and 38 clinical isolates. Table S1: Primers and probes used in the Chinese Entry‐Exit Inspection and Quarantine Industry Standard (SN/T4104‐2015). Table S2: Seven candidate targets identified from comparative genomics analysis of M. hyopneumoniae wild‐type strain 168 and live‐attenuated vaccine strain 168‐L. Table S3: Specific primers targeting selected candidate genes for amplification of M. hyopneumoniae live‐attenuated vaccine strain RM48. Table S4: Homology comparison results of candidate genes across all 18 fully assembled M. hyopneumoniae genomes published in NCBI and RM48. Table S5: The information about the 38 clinical isolates of M. hyopneumoniae. [file TBED-2026-5823134-s001.docx]

**Supplementary material**

Genomic DNA of clinical isolates (n=38) was detected by qPCR method following the Chinese Entry-Exit Inspection and Quarantine Industry Standard (SN/T4104-2015) (Table S1). Genomic DNA of strain 168 served as positive control, genomic DNA of *Actinobacillus pleuropneumoniae* served as negative control, and enzyme-free sterile water was used as the blank control. The qPCR reaction mixture (20 µL) consisted of 10 µL 2 × Premix ExTaq, 0.4 µL of each MHP_SN-F/R primer (10 µM), 0.8 µL MHP_SN-P (10 µM), 0.4 µL Rox Reference DyeII, 5 µL template, and 3 µL enzyme-free sterile water. The cycling conditions were set as follows: initial denaturation at 95 ℃ for 3 min, followed by 40 cycles of denaturation at 95 ℃ for 15 s and annealing at 55 ℃ for 45 s. Amplification results were plotted using GraphPad Prism 10 (Figure S1).

Table S1. Primers and probes used in the Chinese Entry-Exit Inspection and Quarantine Industry Standard (SN/T4104-2015).

| Primer | Sequences (5’ - 3’) | Amplification size (bp) |
| --- | --- | --- |
| MHP_SN-F | CGGAAATTCCTTCCTTTA | 74 |
| MHP_SN-R | TCAGGGTTAATATCAATAATTC |  |
| MHP_SN-P | FAM-AAGTCCTTGATTCATTGCTGC-TAMRA |  |


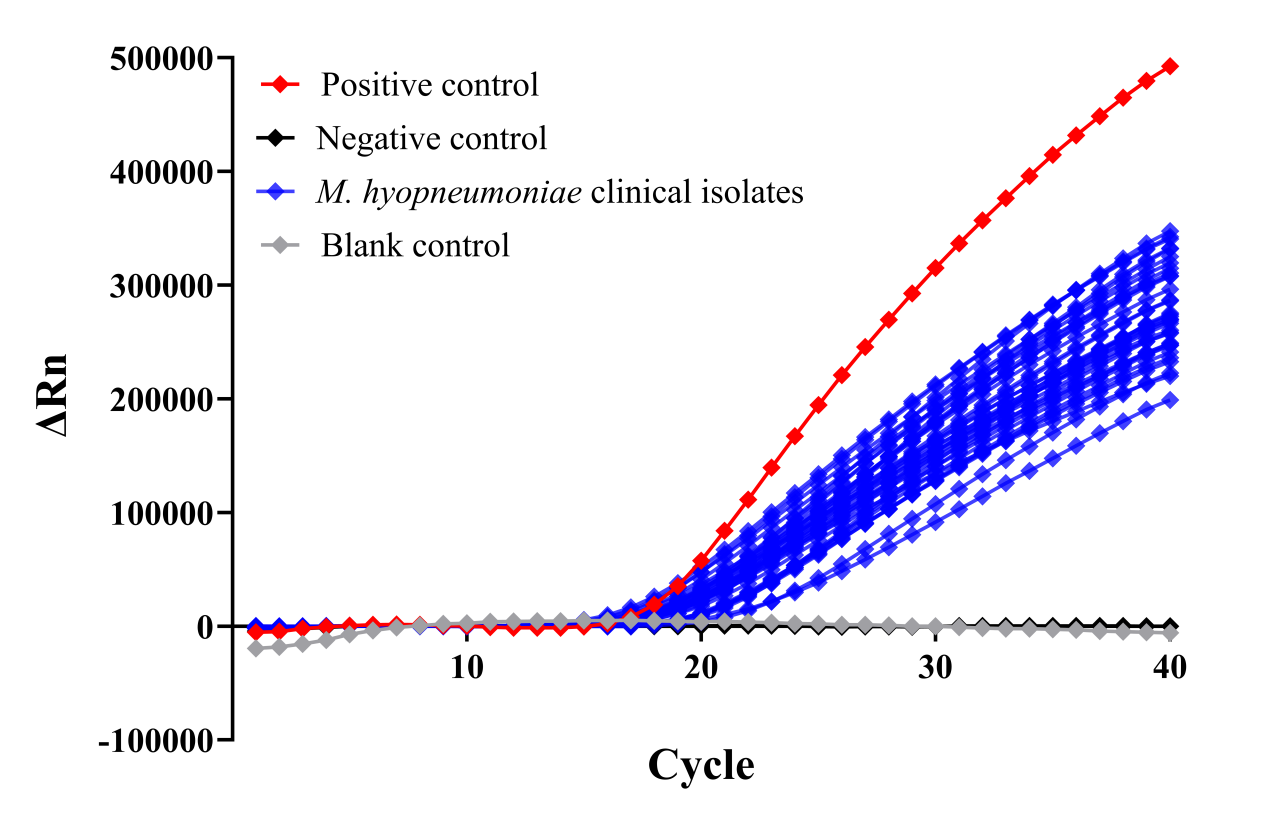


Figure S1. Diagnosis of clinical isolates in accordance with the Chinese Entry-Exit Inspection and Quarantine Industry Standard (SN/T4104-2015).

Whole-genome comparison between the wild-type parental strain 168 and the live-attenuated vaccine strain 168-L was performed, and differential genes harboring insertion sequences longer than 10 bp were screened out as candidate targets for the differential detection of live-attenuated vaccine strain (Table S2).

Table S2. Seven candidate targets identified from comparative genomics analysis of *M. hyopneumoniae* wild-type strain 168 and live-attenuated vaccine strain 168-L.

| 168-L gene | Variation | 168 Locus |
| --- | --- | --- |
| MHP168L_066 | Insertion + SNV | MHP168_066 |
| MHP168L_312 | Insertion + SNV | MHP168_312 |
| MHP168L_314 | Insertion + deletion | MHP168_314 |
| MHP168L_322 | Insertion | MHP168_322 |
| MHP168L_381 | Insertion | MHP168_381 |
| MHP168L_454 | Insertion + SNV | MHP168_454 |
| MHP168L_747 | Insertion + deletion + SNV | MHP168_091 |

For the amplification of *M. hyopneumoniae* live-attenuated vaccine strain RM48, specific primers (Table S3) targeting the seven candidate target genes were designed using Primer Premier 6.0 software and synthesized by General Biology Co., Ltd. The obtained amplification products were sent to Novogene Co., Ltd. for sequencing.

Table S3. Specific primers targeting selected candidate genes for amplification of *M. hyopneumoniae* live-attenuated vaccine strain RM48.

| Primer | Sequences (5’ - 3’) | Amplification size (bp) |
| --- | --- | --- |
| MHP_066-F | CTCATTTCACAAGCCTACAA | 3,235 |
| MHP_066-R | AACCTAGCTCAAGAAGTTCT |  |
| MHP_312-F | GCCTTGCGTTTGTTTAAC | 1,333 |
| MHP_312-R | TCTTCTTCCTCTTGAACTCA |  |
| MHP_314-F | TCTCATCAAACTGGGAAACT | 433 |
| MHP_314-R | CGTGCCGATCACAGTATT |  |
| MHP_322-F | CCAATTCCAGATCTTCCAAT | 1,298 |
| MHP_322-R | AGCAACTAATGGCGAAA |  |
| MHP_381-F | CGGGAGTTTCTTCACTTCTT | 1,986 |
| MHP_381-R | CGAGCTTACTAACATTGACAG |  |
| MHP_454-F | CAAGCTGCGGCAACAA | 2,306 |
| MHP_454-R | AACCCAGGCAGGCAAA |  |
| MHP_747-F | CCCGCAGAAACAAGTTTA | 1,743 |
| MHP_747-R | CAGGCTACTATCGGCTTT |  |

The homology between the seven candidate target genes of strain RM48 and all fully assembled *M. hyopneumoniae* genomes (n=18) deposited in the National Center for Biotechnology Information (NCBI) database was compared using TBTools-II v2.331 and SnapGene ®6.0.2. The MHP168L_314 gene of the live-attenuated vaccine strain 168-L does not share 100% identity with the corresponding genes in other strains. Except for *M. hyopneumoniae* strains J, ES-2 and LH, which exhibited 97.73% sequence identity to MHP168L_314 gene, no homologous sequences of this gene were detected in the genomes of other wild-type strains (Table S4).

Table S4. Homology comparison results of candidate genes across all 18 fully assembled *M. hyopneumoniae* genomes published in NCBI and RM48

| Genes  Strains | MHP168L_066 | MHP168L_312 | MHP168L_314 | MHP168L_322 | MHP168L_381 | MHP168L_454 | MHP168L_747 |
| --- | --- | --- | --- | --- | --- | --- | --- |
| 168-L | 100.00% | 100.00% | 100.00% | 100.00% | 100.00% | 100.00% | 100.00% |
| RM48 | 100.00% | 100.00% | 100.00% | 100.00% | 100.00% | 100.00% | 100.00% |
| 168 | 100.00% | NF | NF | 98.00% | 100.00% | NF | 100.00% |
| 232 | 100.00% | 99.51% | NF | 98.25% | 96.65% | 97.62% | 100.00% |
| 7422 | 100.00% | 98.53% | NF | NF | 98.85% | 97.62% | 97.46% |
| 7448 | 100.00% | 98.53% | NF | NF | 98.85% | 97.62% | 100.00% |
| ES-2 | 100.00% | 99.76% | 97.73% | 96.43% | 98.85% | 97.62% | 100.00% |
| ES-2L | 100.00% | 99.27% | NF | 100.00% | 99.04% | 100.00% | 97.46% |
| F7.2C | 100.00% | NF | NF | 100.00% | 98.66% | 97.62% | 96.61% |
| J | 100.00% | NF | 97.73% | 96.43% | 99.81% | 97.62% | 97.46% |
| KM014 | 100.00% | 98.53% | NF | 100.00% | 98.47% | NF | 100.00% |
| LH | 100.00% | 100.00% | 97.73% | 96.43% | 99.23% | 97.62% | 97.46% |
| 116 | 100.00% | NF | NF | 96.43% | 95.53% | 97.62% | 100.00% |
| 1257 | 100.00% | NF | NF | NF | 98.70% | 97.62% | 100.00% |
| 4284 | 100.00% | NF | NF | 96.43% | 96.26% | 97.62% | 100.00% |
| 15.4B | 100.00% | NF | NF | NF | 98.85% | 97.62% | 100.00% |
| 15.3B | 100.00% | NF | NF | NF | 98.85% | 97.62% | 100.00% |
| 13.3A | 100.00% | 99.51% | NF | 96.43% | 99.42% | 97.62% | 100.00% |
| 13.1B | 100.00% | 99.51% | NF | 96.43% | 99.42% | 97.62% | 100.00% |
| 11.1A | 100.00% | NF | NF | 96.43% | 99.81% | 97.62% | 100.00% |

Abbreviation: NF, Not Found in the whole genome.

Genomic DNA of clinical isolates (n=38) was uploaded to the PubMLST database (http://pubmlst.org). Allele numbers for the target loci (*adk*, *rpoB*, and *tpiA*) were identified using the platform’s BLAST-based query tool. Sequence types (STs) were subsequently assigned according to the database’s classification scheme (Table S5). The MLST results showed that isolates AH-F21 and WX-F14 shared identical allele profiles: *adk* (16),*rpoB* (15), and*tpiA* (34). These profiles were completely consistent with those of the live-attenuated vaccine reference strain 168-L, and all three strains were classified into sequence type 61 (ST61). This result indicates that MLST genotyping exhibits limited discriminatory accuracy in distinguishing between existing *M. hyopneumoniae* vaccine strains and wild-type strains.

Table S5. The information about the 38 clinical isolates of *M. hyopneumoniae*.

| Croup | ID | Country and region of herd | adK | rpoB | tpiA | ST |
| --- | --- | --- | --- | --- | --- | --- |
|  | 168-L | / | 16 | 15 | 34 | 61 |
| Group1 | AH-F21 | Anhui, China | 16 | 15 | 34 | 61 |
|  | WX-F14 | Jiangsu, China | 16 | 15 | 34 | 61 |
|  | AH-2020-6 | Anhui, China | 50 | 58 | 15 | 182 |
|  | GD-2020-18 | Guangdong, China | 51 | 15 | 57 | 184 |
|  | GD-2020-21 | Guangdong, China | 16 | 29 | 56 | 34 |
| Group2 | GD-2020-22 | Guangdong, China | 23 | 23 | 29 | 185 |
|  | GX-2020-17 | Guangxi, China | 23 | 15 | 57 | 128 |
|  | GX-2023-34 | Guangxi, China | 23 | 15 | 59 | 197 |
|  | GX-2023-35 | Guangxi, China | 52 | 15 | 59 | 147 |
|  | GX-2023-36 | Guangxi, China | 52 | 15 | 59 | 147 |
| Group3 | JS-258 | Jiangsu, China | 50 | 58 | 15 | 182 |
|  | JS-266 | Jiangsu, China | 50 | 58 | 15 | 182 |
|  | JS-268 | Jiangsu, China | 50 | 58 | 15 | 182 |
|  | JS-307 | Jiangsu, China | 50 | 58 | 15 | 182 |
|  | JS-787 | Jiangsu, China | 50 | 58 | 15 | 182 |
| Group4 | JS-2019-1 | Jiangsu, China | 52 | 15 | 74 | 187 |
|  | JS-2019-2 | Jiangsu, China | 52 | 15 | 74 | 187 |
|  | JS-2019-3 | Jiangsu, China | 52 | 15 | 74 | 187 |
|  | JS-2019-20 | Jiangsu, China | 52 | 15 | 74 | 187 |
|  | JS-2020-4 | Jiangsu, China | 23 | 59 | 57 | 128 |
| Group5 | JS-2020-5 | Jiangsu, China | 50 | 58 | 15 | 182 |
|  | JS-2020-7 | Jiangsu, China | 50 | 58 | 15 | 182 |
|  | JS-2020-8 | Jiangsu, China | 50 | 58 | 15 | 182 |
|  | JS-2020-9 | Jiangsu, China | 50 | 58 | 15 | 182 |
|  | JS-2020-10 | Jiangsu, China | 16 | 15 | 76 | 186 |
| Group6 | JS-2020-11 | Jiangsu, China | 16 | 1 | 65 | 169 |
|  | JS-2020-13 | Jiangsu, China | 50 | 58 | 15 | 182 |
|  | JS-2020-14 | Jiangsu, China | 50 | 58 | 15 | 182 |
|  | JS-2020-15 | Jiangsu, China | 50 | 58 | 15 | 182 |
|  | JS-2020-16 | Jiangsu, China | 16 | 1 | 65 | 169 |
| Group7 | JS-2020-24 | Jiangsu, China | 54 | 18 | 58 | 130 |
|  | JS-2022-25 | Jiangsu, China | 42 | 15 | 58 | 159 |
|  | JS-2022-26 | Jiangsu, China | 42 | 15 | 58 | 159 |
|  | JS-2022-27 | Jiangsu, China | 42 | 15 | 58 | 159 |
|  | JS-8408 | Jiangsu, China | 50 | 58 | 15 | 182 |
| Group8 | JS-8426 | Jiangsu, China | 50 | 58 | 15 | 182 |
|  | JS-8486 | Jiangsu, China | 50 | 58 | 15 | 182 |
|  | JS-C1 | Jiangsu, China | 6 | 65 | 77 | 4 |

To evaluate the stability of the SNV locus, we examined the target region within the MHP168L_314 gene based on the whole-genome sequences of the 38 *M. hyopneumoniae* clinical isolates included in this study. Sequence comparison confirmed that the 38 *M. hyopneumoniae* clinical isolates possessed adenine (A) at the SNV site, while the live-attenuated vaccine strains 168-L and RM48 contained cytosine (C) (Figure S2).


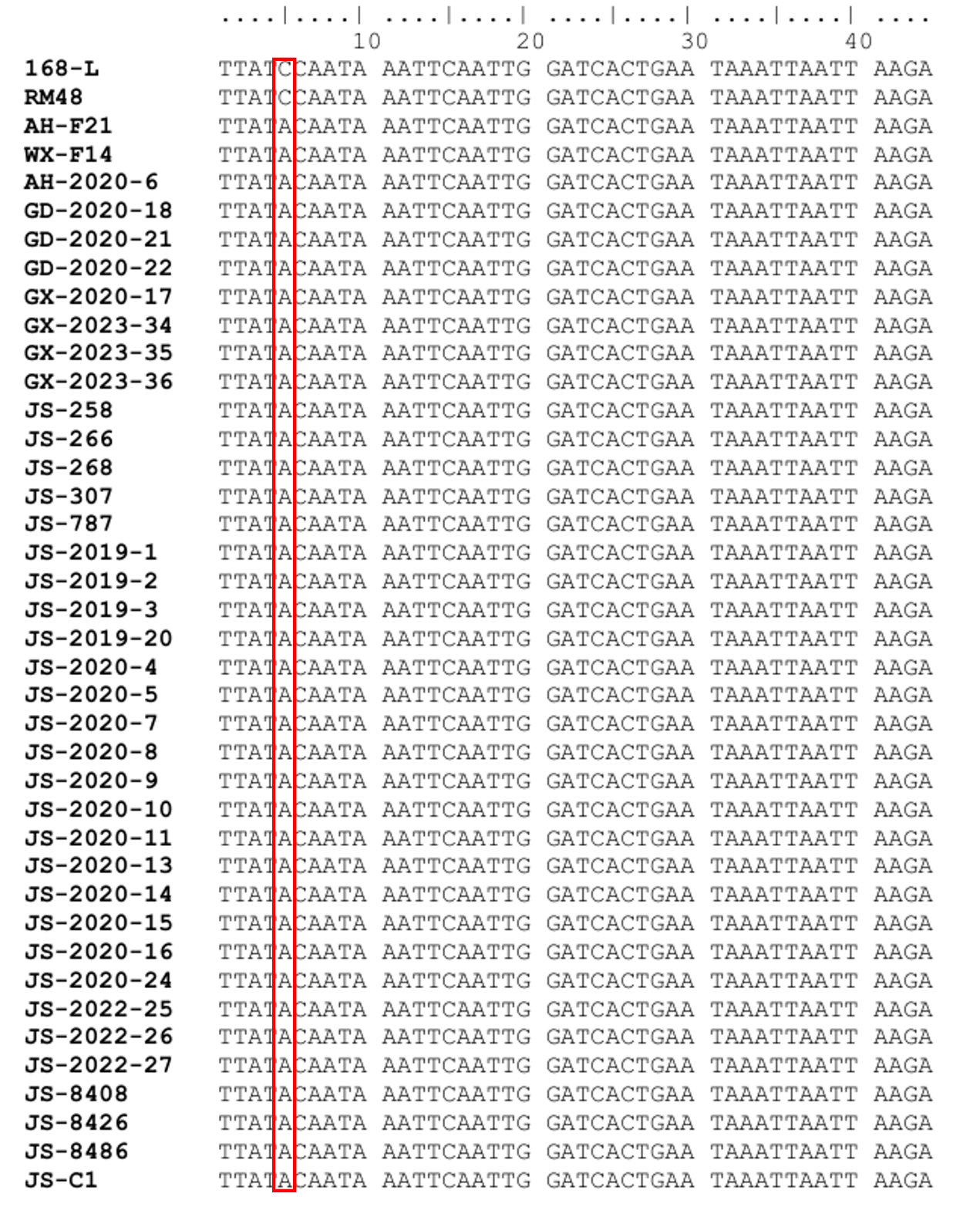


Figure S2. Multiple sequence alignment of the 44 bp target region within the MHP168L_314 gene among *M. hyopneumoniae* live-attenuated vaccine strains and 38 clinical isolates. The SNV site targeted by the RNase H2-linked qPCR assay is highlighted. Live-attenuated vaccine strains contain cytosine (C) at this position, whereas all clinical isolates possess adenine (A).
